# Beneficial and Detrimental Roles of NLRs in Carcinogenesis

**DOI:** 10.3389/fimmu.2013.00370

**Published:** 2013-11-12

**Authors:** Ann M. Janowski, Ryan Kolb, Weizhou Zhang, Fayyaz S. Sutterwala

**Affiliations:** ^1^Inflammation Program, University of Iowa Carver College of Medicine, Iowa City, IA, USA; ^2^Graduate Program in Immunology, University of Iowa Carver College of Medicine, Iowa City, IA, USA; ^3^Department of Pathology, University of Iowa Carver College of Medicine, Iowa City, IA, USA; ^4^Department of Internal Medicine, University of Iowa Carver College of Medicine, Iowa City, IA, USA; ^5^Veterans Affairs Medical Center, Iowa City, IA, USA

**Keywords:** inflammasomes, NLR, cancer, interleukin-1, interleukin-18

## Abstract

Inflammation plays a critical role in tumorigenesis and can contribute to oncogenic mutations, tumor promotion, and angiogenesis. Tumor-promoting inflammation is driven by many factors including the presence of the pro-inflammatory cytokines interleukin (IL)-1β and IL-18. One major source of IL-1β and IL-18 secretion is through the activation of inflammasomes. Inflammasomes are multi-protein complexes that upon activation lead to the processing and secretion of IL-1β and IL-18 mediated by the cysteine protease caspase-1. Several inflammasomes, including NLRP3, NLRC4, and NLRP6, have been implicated in tumorigenesis. However, inflammasomes play divergent roles in different types of cancer reflecting the complexity of inflammation during tumorigenesis. Understanding the role of inflammasome activation during specific stages of tumorigenesis and also during cancer immunotherapy will help identify novel therapeutic targets that could improve treatment strategies for cancer patients. Here we will discuss recent advances in understanding the mechanism by which NLRs regulate carcinogenesis.

## Introduction

In order to protect us from infection, the immune system has employed an arsenal of pattern recognition receptors (PRRs) capable of recognizing a wide variety of microbial pathogens and viruses. PRRs are also activated under conditions of cell stress through the detection of damage associated molecular patterns (DAMPs) (1, 2). However, it is not well understood if and how the immune system has evolved to recognize overexpressed or mutated self-antigens in the context of cancer. Various PRRs have been implicated in cancer, either employing a protective or detrimental role. However, it remains unknown what causes the activation or suppression of PRRs within the tumor microenvironment. The activation of PRRs by tumor-related signals, in many cases, leads to the release of pro-inflammatory cytokines which can be beneficial by leading to proper activation of antigen presenting cells and subsequent T cell activation. However, it is well established that chronic inflammation can play roles during all stages of tumorigenesis and can be associated with poor clinical prognosis, depending on the type of pro-inflammatory cytokines and cancer (3–5). Here we describe the current literature on the role of PRRs, with a special focus on nucleotide-binding leucine-rich repeat (NLR) proteins, in cancer and how they might be utilized in tumor immunotherapy (summarized in Table 1).

**Table 1:** **Role of NLRs in cancer**.

NLR	Type of cancer	Possible mechanism	Reference
NLRC4	Plays a protective role in mouse models of colitis-associated colorectal cancer (CAC)	NLRC4 may regulate apoptosis in colonic epithelial cells	Hu et al. (6), and Sadasivam et al. (7), and Hu et al. (8)
NLRP6	Plays a protective role in CAC	Absence of NLRP6 leads to an increase in *Prevotellaceae* in the intestines and increased inflammation	Elinav et al. (9), Hu et al. (10), Normand et al. (11), and Chen et al. (12)
NLRP3	Plays a protective role in CAC	NLRP3 drives secretion of IL-18 which leads to activation of STAT1 and tumor suppression	Allen et al. (13) and Zaki et al. (14)
	Promotes tumor formation in a mouse model of fibrosarcoma	Presence of NLRP3 leads to an decrease in NK cells and CD11b^+^Gr-1^int^ myeloid cells leading to increased tumorigenesis	Chow et al. (15)
	Promotes pulmonary metastasis in intravenous B16F10 and RM-1 prostate mouse models		
NLRP12	Plays a protective role in CAC	Absence of NLRP12 leads to dysregulation of canonical or non-canonical NF-κB signaling leading to increased inflammation	Zaki et al. (16) and Allen et al. (17)

The NLR family is comprised of over 22 members in humans (18, 19). NLR proteins are characterized by the presence of three homologous domains: a central nucleotide-binding and oligomerization (NACHT) domain, a C-terminal leucine-rich repeat domain (LRR), and an N-terminal effector domain (18, 19). As the name indicates, the NACHT domain is important for oligomerization, the LRR domain is important for ligand sensing, and the N-terminal effector domain recruits downstream signaling molecules (20, 21).

Within the NLR family, NLRP1, NLRP3, and NLRC4, as well as the PYHIN family member Absent in Melanoma 2 (AIM2), have been shown to form large multi-protein complexes termed inflammasomes. There is evidence that NLRP6 may also form a functional inflammasome (9); however, additional studies are required to confirm this. Inflammasomes are composed of an NLR protein (or AIM2), an adaptor protein apoptosis-associated speck-like protein containing a Card domain (ASC), and the cysteine protease caspase-1. Inflammasome activation is a two-step process requiring a priming step and an assembly step. The priming step results in the transcription of pro-IL-1β and pro-IL-18 along with certain inflammasome components (22, 23). The second step, which can be triggered by a variety of stimuli, results in the assembly and activation of inflammasomes. Inflammasome activation leads to the cleavage of pro-caspase-1 and its subsequent activation, which in turn cleaves pro-IL-1β and pro-IL-18 into their mature forms that can be secreted from the cell. Additionally, caspase-1 activation can lead to an inflammatory form of cell death known as pyroptosis (18, 23).

In addition, there are also reports of non-canonical inflammasome activation independent of caspase-1. For example, the receptor dectin-1 activates caspase-8 leading to processing and secretion of IL-1β upon sensing fungi or mycobacteria (24). Non-canonical inflammasome activation of caspase-11 has also been shown to be important for IL-1β secretion, caspase-1 activation, and macrophage cell death in response to *Escherichia coli, Citrobacter rodentium*, and *Vibrio cholera* (25). These data demonstrate important roles of various other pathways in processing of IL-1β. Importantly, non-canonical inflammasomes may also play a role in cancer-related inflammation.

Within the past 10 years, cancer-related inflammation was added to the list of cancer hallmarks (26, 27). Inflammation has been linked to tumor initiation, progression, angiogenesis, metastasis, tumor cell proliferation and survival, and alterations in the anti-tumor adaptive immune response. Sources of tumor-related inflammation include bacterial and viral infections, environmental irritants and obesity, and tumor-elicited or therapy-induced inflammation. Pro-inflammatory cytokines, such as IL-1β and IL-6, are critical mediators for inflammation-promoted tumorigenic effects and play critical roles in tumorigenesis (3, 4, 26, 27). For future development of therapeutic strategies, it will be crucial to understand how NLRs and inflammasomes are activated during tumor progression and how pro-inflammatory cytokines downstream of their activation impact tumorigenesis.

## NLRC4

### NLRC4

**Figure 1:**
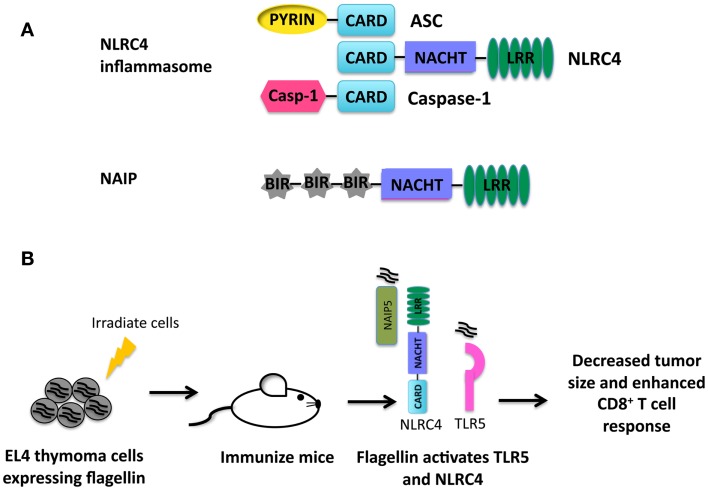
**(A)** Structure of the NLRC4 inflammasome and NAIP proteins. **(B)** Summary of immunotherapy utilizing activation of NLRC4 (43). EL4 thymoma cells were transduced to express flagellin from *S. typhimurium*. Mice were then immunized with irradiated EL4 cells expressing flagellin. Flagellin is recognized by TLR5 and NLRC4 in the presence of tumor antigens leading to production of pro-inflammatory cytokines and an enhanced anti-tumor CD8^+^ T cell response.

The PRR NLRC4 contains an N-terminal caspase activation and recruitment domain (CARD), a central NACHT domain, and a C-terminal LRR as depicted in Figure 1A (28, 29). The CARD domain present in NLRC4 allows for direct interaction with pro-caspase-1 (28). However, optimal caspse-1 activation requires the adaptor protein ASC (30–32). NLRC4 is classically understood to recognize a number of Gram-negative bacteria including *Salmonella enterica, Anaplasma phagocytophilum, Pseudomonas aeruginosa*, and *Legionella pneumophila*, which in turn leads to subsequent activation of the NLRC4 inflammasome (31, 33–35). More specifically, cytosolic flagellin and proteins with structural homology to flagellin such as PrgJ, a component of type III secretion systems, have also been shown to activate the NLRC4 inflammasome (36–38). The NLRC4 inflammasome works in concert with neuronal apoptosis inhibitor protein (NAIP) 2 to recognize PrgJ-like proteins and NAIP5 to recognize cytosolic flagellin (39–41). NAIP proteins, like NLRC4, have a C-terminal LRR domain, a central NACHT domain, and an N-terminal baculovirus IAP repeat (BIR) instead of the CARD domain (42).

### NLRC4 and cancer

NLRC4 along with caspase-1 has been shown to regulate tumorigenesis in a mouse model of colitis-associated colorectal cancer (CAC) (6). In this model, cancer was induced by administration of azoxymethane and dextran sodium sulfate (AOM-DSS) to mice. Interestingly, *Nlrc4*^−/−^ and *caspase-1*^−/−^ mice exhibited increased tumor formation compared to wild-type mice. However, this phenotype was specific to colorectal cancer and not DSS colitis indicating that increased colonic inflammation was not the driving force for the enhanced tumorigenesis seen in *Nlrc4*^−/−^ and *caspase-1*^−/−^ mice and instead was likely due to a cell intrinsic mechanism. Although a definitive mechanism was not determined in the study, it is speculated that NLRC4 may be playing a role in alterations in colonic epithelial cell apoptosis as p53 activation has been linked to NLRC4 gene expression (7). Such a notion was further supported by the generation of wild-type and *Nlrc4*^−/−^ bone marrow chimeras. *Nlrc4*^−/−^ mice receiving a wild-type bone marrow transplant had similar tumor loads as *Nlrc4*^−/−^ mice receiving *Nlrc4*^−/−^ bone marrow in the CAC model, but exhibited significantly higher tumor burdens than wild-type mice receiving either wild-type or *Nlrc4*^−/−^ bone marrow (8). This observation indicates that NLRC4 plays a CAC-suppressive role confined to cells other than in the hematopoietic compartment.

An alternative hypothesis that has been proposed involves the role of NLRC4 in the regulation of commensal microbiota. NLRC4 is important for detecting bacterial pathogens in the intestines without becoming activated by the presence of commensal organisms (44). Interestingly, *Nlrc4*^−/−^ mice have alterations in their microflora compared to wild-type mice (9), indicating that NLRC4 is in some way important for the regulation of intestinal microbiota. The differences in microbiota seen in *Nlrc4*^−/−^ mice did not lead to enhanced susceptibility to DSS-induced colitis (9). In concert with these data *Nlrc4*^−/−^ mice co-housed with wild-type mice, which leads to transmission of the intestinal microbiota, exhibited no differences in CAC tumorigenesis (10). Therefore indicating that alterations in the microbiota in the absence of NLRC4 are not correlated with progression of CAC.

### NLRC4 and tumor immunotherapy

Activation of NLRC4 results in the potent secretion of pro-inflammatory cytokines necessary for priming an effective adaptive immune response. Priming an immune response is especially problematic in a tumor environment where antigens are altered or are simply overexpressed self-antigens and the immune milieu is generally suppressive (45). A novel set of experiments were performed integrating the activation of TLR5, NAIP5, and NLRC4 by bacterial flagellin into tumor immunotherapy (43). In these studies, B16 melanoma cells and EL4 thymoma cells expressing flagellin from *Salmonella typhimurium* were generated. Both EL4 and B16 cells expressing flagellin were unable to establish tumors *in vivo*. In addition, EL4 and B16 cells expressing flagellin also induced a potent anti-tumor response from CD4 and CD8 T cells. Immunization of mice with irradiated EL4 cells expressing flagellin protected mice during subsequent challenge with live EL4 cells (Figure 1B). These data indicate that activation of TLR5, NAIP5, and NLRC4 during therapeutic strategies including tumor cell vaccination could be beneficial.

## NLRP6

### NLRP6

**Figure 2:**
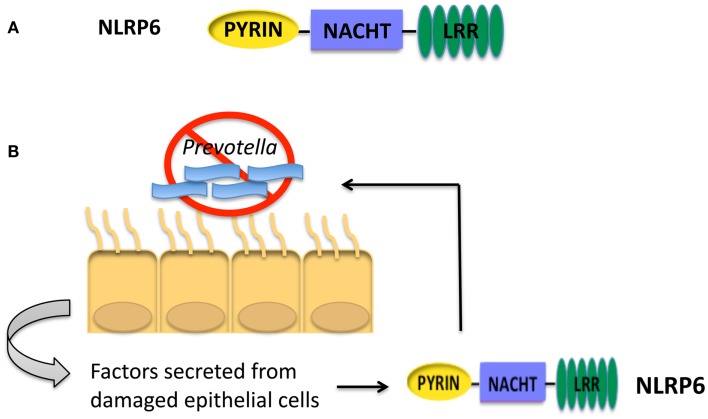
**(A)** Structure of NLRP6. **(B)** NLRP6 is important for controlling the growth of *Prevotella* in the intestines of mice (9). Interactions between intestinal epithelial cells and pathogenic species of microbiota will lead to epithelial cell damage. Factors secreted from damaged epithelial cells activate NLRP6. Activation of NLRP6 leads to processing and secretion of IL-18 and a decrease in *Prevotella*. The presence of *Prevotella* is correlated with colitis and colorectal cancer in humans.

The structure of NLRP6 is comprised of an N-terminal Pyrin domain, a central NACHT domain and a C-terminal LRR as seen in Figure 2A (46). It is unclear if NLRP6 is able to form a functional inflammasome like NLRP1, NLRP3, NLRC4, or AIM2. From overexpression studies in human embryonic kidney (HEK) 293T cells, NLRP6 was recruited to ASC speck-like structures. In addition, COS-7L cells co-transfected with plasmids encoding pro-caspase-1, ASC, and NLRP6 were shown to secrete IL-1β (46). Additionally, *in vivo* studies suggest that NLRP6 may be forming an inflammasome (9). However, whether NLRP6 is able to be recruited to an inflammasome complex and cleave IL-1β when expressed at basal levels has not been determined.

NLRP6 has no identified ligand; however, it was recently shown to play a significant role as a negative regulator of inflammatory signaling during bacterial infection (47). *Nlrp6*^−/−^ mice had decreased mortality and bacterial burdens when challenged with *Listeria monocytogenes* and *S. typhimurium*, accompanied by increased neutrophil influx. Additionally, *Nlrp6*^−/−^ macrophages had increased NF-κB and ERK activation in response to bacterial infection. These data highlight NLRP6 as a negative regulator of inflammation with a role to potentially dampen pathology and damage to the host.

### NLRP6 and cancer

NLRP6 is highly expressed in the duodenum, ileum, and colon, which prompted studies to determine the role of NLRP6 in the intestine (11). Like NLRC4, NLRP6 was shown to negatively regulate colitis and CAC in mice (9–12). In the AOM-DSS CAC model, *Nlrp6*^−/−^ mice had increased pathology and tumor numbers compared to wild-type mice. *Nlrp6*^−/−^ colons exhibited increased expression of pro-inflammatory cytokines indicating an inability of mice to control inflammation within the intestine. Moreover, *Nlrp6*^−/−^ mice exhibited an increased level of epithelial proliferation and a decreased ability to heal wounds (11, 12). These data demonstrate a unique role for NLRP6 in controlling intestinal inflammation, preserving the integrity of the intestinal epithelial barrier, and wound healing in the intestine. Without NLRP6, the intestine is more susceptible to epithelial damage, which in turn increases inflammation and susceptibility to CAC.

In other studies focusing on the role of NLRP6 and colitis, *Nlrp6*^−/−^ mice were shown to have significantly altered intestinal microbiota (9). Of note, *Nlrp6*^−/−^ mice had an increase in the phylum Bacteroidetes family *Prevotellaceae* as described in Figure 2B. The increased inflammation and colitis seen in *Nlrp6*^−/−^ mice was attributed to the presence of *Prevotellaceae*, and these colitis-conferring bacteria could be transmitted to co-housed wild-type mice leading to enhanced disease. The role of *Prevotellaceae* was not examined in the mouse model of CAC. However, wild-type mice co-housed with *Nlrp6*^−/−^ mice exhibit similar tumor score indicating that the microbiota present in *Nlrp6*^−/−^ mice promotes enhanced CAC (10). In addition, due to the increased inflammation present in the intestine colonized with *Prevotellaceae*, it could be hypothesized that the presence of *Prevotellaceae* might lead to an increased susceptibility to colorectal cancer. Interestingly, *Prevotellaceae* was also shown to be prominent in the microbiota of patients with inflammatory bowel disease and was significantly increased in colorectal cancer patients (48–50), thus providing a link between *Prevotellaceae* and colorectal cancer. However, it is unknown whether the increased inflammation due to presence of *Prevotellaceae* leads to inflammatory bowel disease and subsequent increased risk for colorectal cancer, or if *Prevotellaceae* can directly increase the risk for colorectal cancer independent of inflammatory bowel disease. Additional studies need to be performed to clarify the role of *Prevotellaceae* in the development of colorectal cancer.

### NLRP6 and tumor immunotherapy

Although there is no described tumor immunotherapy directly involving NLRP6, this area of research represents an interesting potential. As mentioned above, NLRP6 is a negative regulator of inflammation during bacterial infections. However, the role of NLRP6 as a negative regulator of sterile inflammation has not been explored. Chronic inflammation plays a crucial role in the initiation and progression of cancer (3, 4). If NLRP6 has a more global role in suppressing inflammation, it is possible that NLRP6 may be important in dampening tumor-promoting inflammation. Alternatively, inflammation is important for activating dendritic cells for proper antigen presentation and T cell activation (45). NLRP6 has the potential to alleviate inflammation that is crucial for an effective T cell response. Further studies of NLRP6 will need to be pursued to determine if it contributes to tumor progression or protection.

Alterations in microbiota have been shown to impact the severity of inflammatory bowel disease and colorectal cancer in humans and mice (48–50). The microbiota is very pliable making it an ideal therapeutic target. With the development of probiotics, fecal transplants, and antibiotic treatments, there are a number of ways to treat dysbiosis in the intestine. Preventative treatments to alter the microbiota of patients with inflammatory bowel disease or dysbiosis may decrease levels of inflammation present in the intestine, thereby decreasing the risk of developing colorectal cancer. Moreover, treatment of dysbiosis in colorectal cancer patients may alleviate symptoms. Future studies should include determining if mutations in NLRP6 are correlated with colorectal cancer and dysbiosis in humans, specifically with the increased *Prevotellaceae*. If a correlation exists between NLRP6 and colorectal cancer in humans, NLRP6 may serve as a valuable biomarker and therapeutic target.

## NLRP3

### NLRP3

**Figure 3:**
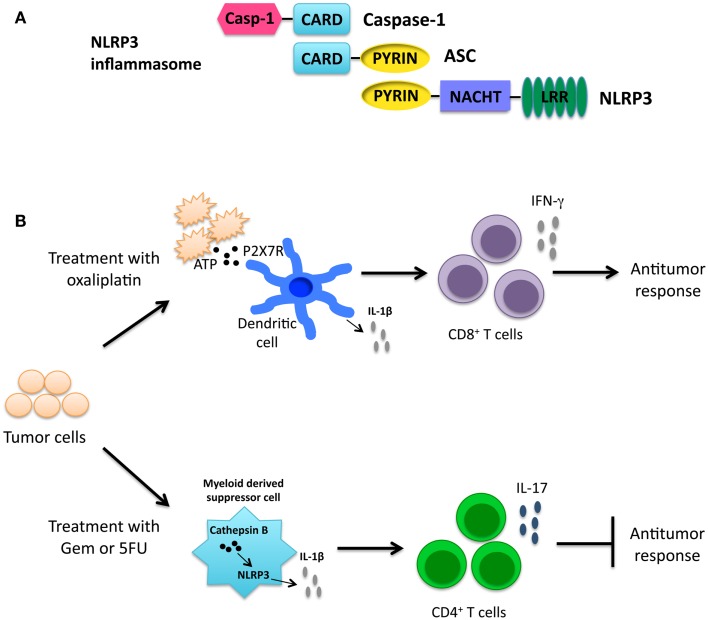
**(A)** Structure of the NLRP3 inflammasome. **(B)** Cancer therapies activating the NLRP3 inflammasome have opposing roles. Treatment with the chemotherapeutic drug oxaliplatin leads to the release of ATP from dying cancer cells. ATP interacts with the P2X7 receptor on dendritic cells leading to activation of the NLRP3 inflammasome. IL-1β secreted by dendritic cells primes anti-tumor CD8^+^ T cells and promotes the anti-tumor response (66). In contrast, gemcitabine (Gem) and 5-fluorouracil (5FU) activate the NLRP3 inflammasome via release of cathepsin B in myeloid derived suppressor cells leading to IL-1β secretion. IL-1β promotes CD4^+^ T cells to secrete the cytokine IL-17, which blunts the anti-tumor response (67).

NLRP3 contains an N-terminal Pyrin domain, a NACHT domain, and a C-terminal LRR as seen in Figure 3A (18). NLRP3 is expressed by a number of cells including epithelial cells, neutrophils, macrophages, and dendritic cells (51, 52). Upon activation, NLRP3 forms a complex with ASC, and caspase-1 leading to pyroptosis and the release of inflammatory cytokines. A number of stimuli are able to activate the NLRP3 inflammasome. Pathogens including *Candida albicans, Staphylococcus aureus*, and *Influenza*, among others, have been shown to activate the NLRP3 inflammasome (53–55). Additionally, host-derived stress or danger signals are able to activate the NLRP3 inflammasome, including extracellular ATP and monosodium urate crystals (55–57). Exposure to environmental irritants including silica and asbestos will also lead to activation of the NLRP3 inflammasome (58–60). It is hypothesized that the numerous diverse NLRP3 agonists converge on a common pathway that results in NLRP3 inflammasome activation. Currently, potassium and calcium fluxes along with the generation of reactive oxygen species (ROS) and mitochondrial dysfunction have all been shown to be required for NLRP3 inflammasome activation (61–65).

Cancer cells also exhibit altered metabolic activity in order to support increased proliferation and survival, and can sometimes display increased ROS production (68, 69). Thus, it could be hypothesized that dysfunctional mitochondria in cancer cells activate the NLRP3 inflammasome, leading to an increase in tumor-promoting pro-inflammatory cytokines. NLRP3 activation by mitochondria-associated factors from cancer cells is not well studied and is an area that warrants further investigation.

### NLRP3 and cancer

NLRP3, like NLRC4 and NLRP6, is important for prevention of CAC development in the AOM-DSS model (13, 14). *Nlrp3*^−/−^ mice had increased polyp numbers and size and worsened pathology compared to wild-type mice. This phenotype was also seen in *Asc*^−/−^ and *caspase-1*^−/−^ mice, indicating that the NLRP3 inflammasome is important in suppressing CAC development. Importantly, it was shown that the presence of NLRP3 in hematopoietic cells was necessary for this the tumor-suppressing effect in response to AOM-DSS challenge (13). Furthermore, IL-18 levels were dramatically reduced in the colon of *Nlrp3*^−/−^ and *caspase-1*^−/−^ mice. Treatment of *caspase-1*^−/−^ mice with recombinant IL-18 led to a reduction in disease demonstrating a crucial role of IL-18 in protection against CAC development (14). Partial amelioration of disease in *caspase-1*^−/−^ by administration of exogenous IL-18 was also demonstrated in a separate study (70). In concert with these data, *Myd88*^−/−^, *Il18*^−/−^, and *Il18r*^−/−^ mice were also shown to be more susceptible to DSS-induced colitis and colon cancer (71).

IL-18 was proposed to have a role in IFN-γ mediated activation of STAT1, which is known to have a role in tumor suppression (72). These data imply that the absence of NLRP3 results in decreased IL-18 production and STAT1 signaling that are necessary for protection against CAC potentially by playing a role in epithelial repair. Treatment with recombinant IL-18 did not fully alleviate symptoms indicating that some other mechanisms may be at play. It should be noted that in another study utilizing the AOM-DSS model, *Nlrp3*^−/−^ mice exhibited a similar tumor load as WT mice (6). The difference in phenotypes may be attributed to alterations in mouse intestinal microbiota or differences in experimental procedures. However, whether NLRP3 is important for protection in colitis-associated cancer still remains unclear.

In contrast to the protective role of NLRP3 in CAC, NLRP3 was shown to promote tumor formation in a chemical-induced fibrosarcoma model (15). *Nlrp3*^−/−^ mice treated with methylcholanthrene (MCA) exhibited prolonged tumor-free survival compared to wild-type mice. In concert with this data, *Nlrp3*^−/−^ mice challenged intravenously with B16F10 melanoma and RM-1 prostate carcinoma cells had significantly fewer metastasis compared to wild-type mice. Reduced pulmonary metastasis was also seen in *Nlrp3*^−/−^ mice using orthotopic transplant of E0771 mammary adenocarcinoma cells (15). The decrease in tumor formation was attributed to an increase in natural killer (NK) cells and CD11b^+^Gr-1^int^ myeloid cells seen in *Nlrp3*^−/−^ mice. The CD11b^+^Gr-1^int^ myeloid cells secreted CCL5 and CXCL9 that were important for recruiting NK cells into the tumor microenvironment (15), demonstrating a role for NLRP3 in the suppression of NK cell activation and promotion of a suppressive tumor environment.

In an interesting *in vitro* study of late stage metastatic melanoma cell lines, HS294T and 1205Lu cells were shown to constitutively produce IL-1β in culture (73). In contrast, non-metastatic melanoma cell lines secreted less or no IL-1β. HS294T and 1205Lu cells also expressed NLRP3, ASC, and caspase-1 and secreted factors that resulted in increased macrophage chemotaxis and angiogenesis (73). These data merit further study on the role of inflammasome activation in metastatic tumor cells, and how inflammasome activation is altered in tumor cells as they progress from non-metastatic cells to ones with metastatic capacity.

### NLRP3 and immunotherapy

As inflammation has been shown to be both beneficial and detrimental for cancer, it is fitting that NLRP3 activation in immunotherapy can also be beneficial and detrimental. Chemotherapy-induced cell death was shown to result in both priming and activation of the NLRP3 inflammasome. Priming occurred through TLR4 by high mobility group box-1 (HMGB1) protein; ATP released from dying cells then lead to activation of the NLRP3 inflammasome (66). NLRP3 activation in dendritic cells by oxaliplatin-treated tumor cells resulted in the release of IL-1β that was critical for priming of anti-tumor CD8^+^ T cells as depicted in Figure 3B. This knowledge is useful in formulating a therapeutic setting where chemotherapeutic drugs known to activate the NLRP3 inflammasome may be used to improve prognosis.

In contrast, the chemotherapeutic agents gemcitabine (Gem) and 5-fluorouracil (5FU), which have been shown to deplete myeloid derived suppressor cells (MDSCs), activated NLRP3 in MDSCs resulting in a diminished anti-tumor response (67, 74, 75). Treatment with Gem or 5FU resulted in the release of cathepsin B that was shown to activate NLRP3 with the subsequent secretion of IL-1β. IL-1β then led to an enhanced IL-17 production by CD4^+^ T cells as depicted in Figure 3B. Interestingly, treatment of *Il17a*^−/−^ mice with 5FU resulted in decreased tumor size (67). Taken together these findings demonstrate a role for NLRP3 activation in skewing a Th17 response and leading to a decreased anti-tumor response. It should be noted that 5FU treatment combined with IL-1R-blocking antibody lead to decreased tumor size. Although treatment with IL-17-blocking antibodies was not included in this study, combining IL-1R or IL-17 blocking antibodies with chemotherapeutic treatment may lead to a better outcome for some patients.

Another cancer immunotherapy is the use of dendritic cell vaccinations. Interestingly, the presence of NLRP3 led to a decrease in survival during B16F10 melanoma challenge and subsequent vaccination with B16-pulsed dendritic cells (76). The decrease in vaccine efficacy was due to a significant increase in tumor-infiltrating MDSCs and a decrease in CD8^+^ effector T cells. How NLRP3 recruits MDSCs into the tumor environment is unknown. MDSCs are very potent immunosuppressive cells that are associated with increased tumor growth (77). Determining the role that NLRP3, or other NLRs, may play in MDSC recruitment will be an important area of research to explore.

## NLRP12

### NLRP12

**Figure 4:**
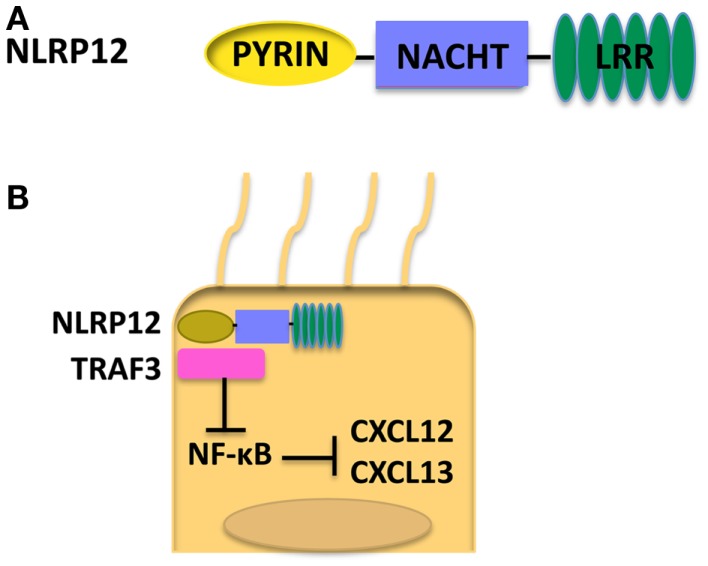
**(A)** Structure of NLRP12. **(B)** When NLRP12 is present in intestinal epithelial cells it interacts with TRAF3 leading to stabilization of TRAF3 levels. Stabilization of TRAF3 leads to regulation of the non-canonical NF-κB pathway and diminished levels of the chemokines CXCL12 and CXCL13, both of which are upregulated in human cancers. Regulation of the non-canonical NF-κB pathway by NLRP12 leads to decreased inflammation and tumorigenesis in the colon (17).

NLRP12, also known as Monarch-1, is composed of an N-terminal Pyrin domain, an NACHT domain, and a C-terminal LRR (78) as shown in Figure 4A. NLRP12 was shown to associate with ASC in an overexpression system; however, whether NLRP12 forms a functional inflammasome has not been well documented (79). NLRP12 is highly expressed in granulocytes in the bone marrow, and also macrophages in the spleen (80). Currently, NLRP12 is viewed as a regulator of inflammation. However, NLRP12 has been shown to have opposing roles in activation of NF-κB. Transient transfection of 293T cells with NLRP12 and ASC constructs led to transcription of an NF-κB luciferase reporter, demonstrating a role for NLRP12 in the activation of NF-κB (79). NLRP12 seems to inhibit the non-canonical NF-κB activation in the human monocytic cell line THP-1 by the binding of NLRP12 to NF-κB inducing kinase (NIK) and leading to the degradation of NIK (81). Interestingly, in humans, mutations in NLRP12 have been associated with a periodic fever syndrome (82, 83). When HEK293T cells were transfected with NLRP12 constructs harboring these mutations, an increase of NF-κB activation was seen (82). Missense mutations in NLRP12 in periodic fever syndrome were also associated with increased caspase-1 activation and had no effect on NF-κB (83, 84). These data further demonstrate NLRP12 as a potential negative regulator of inflammation and imply that NLRP12 may play a causal role in certain human disease.

NLRP12 has also been implicated in the control of bacterial pathogens. Both NLRP12 and IL-18 were shown to be crucial for control of *Yersinia pestis* infection (85), but NLRP12 was dispensable during infections with *Klebsiella pneumoniae* and *Mycobacterium tuberculosis* (80).

### NLRP12 and cancer

The role of NLRs in colorectal cancer is well studied. NLRP12, like NLRP3, NLRP6, and NLRC4, plays a protective role during colorectal tumorigenesis. *Nlrp12*^−/−^ mice exhibited an increase in tumor numbers in the AOM-DSS CAC model. Additionally, colons from *Nlrp12*^−/−^ mice had increased tissue damage, pro-inflammatory cytokine production, and ERK, STAT3, and NF-κB activation (16). The increased tumorigenesis was attributed to a lack of NLRP12 in hematopoietic cells. Bone marrow-derived macrophages from *Nlrp12*^−/−^ mice exhibited an increase in phosphorylation of p105/NF-κB1 in response to lipopolysaccharide stimulation demonstrating a role for NLRP12 in regulation of the canonical NF-κB pathway (16). It was hypothesized that dysregulation of the canonical NF-κB signaling in *Nlrp12*-deficient hematopoietic cells leads to increased inflammation and tumorigenesis.

In a more recent study, NLRP12 expression in non-hematopoietic cells was shown to play a protective role in CAC (17). *Nlrp12*^−/−^ mice exhibited increased inflammation and tumor numbers. Interestingly, both *in vitro* and *in vivo* NLRP12 deficiency resulted in an increased activation of the non-canonical NF-κB pathway. *Nlrp12*^−/−^ mice expressed elevated levels of CXCL12 and CXCL13, and also increased phosphorylation of ERK (17). It was hypothesized that NLRP12 negatively regulates non-canonical NF-κB signaling via interactions with NIK and TRAF3. In the absence of NLRP12, there is an increased level of inflammation and cancer-promoting chemokines as depicted in Figure 4B. Discrepancies seen in canonical versus non-canonical NF-κB signaling *in vitro* may be due to the nature of the stimulus used. NLRP12 was shown to downregulate canonical NF-κB signaling in response to stimulation with TLR agonists whereas, in response to stimulation with TNF-α or CD40L, NLRP12 regulates non-canonical NF-κB signaling. These data suggest NLRP12 may modulate both canonical and non-canonical NF-κB signaling depending on the upstream stimuli.

It is possible that a common pathway exists which NLRs converge on leading to protection against colorectal cancer. This likely includes maintenance of epithelial barrier integrity. Inflammation, as a result of altered pro-inflammatory signaling or altered microbiota, leads to the breakdown of the epithelial barrier in the intestine. When the barrier between immune cells and the microbiota dissolves, PRR ligands are in abundance and activate cells in the intestine, leading to the release of more pro-inflammatory cytokines and creating a cycle of inflammation that leads to development and progression of cancer.

### NLRP12 and immunotherapy

The possibility of targeting NLRP12 as a therapeutic treatment has not been explored. NLRP12 is crucial for the down-regulation of NF-κB signaling. Activation of NF-κB has been correlated with enhanced tumor cell survival and growth (86). As NLRP12 is a regulator of NF-κB, it would be beneficial to determine the role of NLRP12 in human cancer. Additionally, it may serve as a more specific target in immunotherapy compared to NF-κB.

## Interleukin-1β

### IL-1β and cancer

As mentioned above, inflammasome activation leads to the processing and secretion of IL-1β. IL-1β is a potent pro-inflammatory cytokine associated with tumor growth and angiogenesis (87). In a study utilizing a B16 melanoma model, IL-1β-deficient mice had remarkably reduced subcutaneous tumor size and lung metastasis compared to wild-type mice. Additionally, IL-1β-deficient mice and wild-type mice treated with an IL-1R blocking antibody had significantly reduced angiogenesis as measured by microvessel density compared to wild-type mice (88). In concert with these data, IL-1β- and IL-1α-deficient mice had decreased tumor numbers and tumor size in the MCA model of fibrosarcoma. Additionally, IL-1R antagonist (IL-1Ra)-deficient mice had increased tumor size (89). Expression of IL-1β by tumor cells also contributes to tumor growth and angiogenesis. Mice challenged with Lewis lung carcinoma cells (LLC) transduced with human IL-1β exhibited increased tumor size and vasculature compared to LLC cells alone (90). These studies are critical in demonstrating a role for IL-1β in promoting growth of solid tumors, angiogenesis, and metastasis.

### IL-1β and immunotherapy

IL-1 expression is enhanced in a number of cancers including lung, colon, melanoma, and breast (91, 92). Studies in mouse models have demonstrated therapeutic promise for treatment with IL-1 blocking antibodies and also combining IL-1 blocking antibodies with traditional anti-cancer immunotherapies (67, 87, 88). Anakinra is an IL-1R antagonist that is used to treat a number of autoinflammatory disorders (91). Anakinra in combination with dexamethasone has shown promise in slowing myeloma proliferation in patients with smoldering myeloma (93). Due to the strong correlation between IL-1β and tumor progression, it is worthwhile to continue to explore Anakinra and other IL-1R antagonists as tumor immunotherapy options.

## Interleukin-18

### IL-18 and cancer

Along with IL-1β, the cytokine IL-18 is also cleaved by caspase-1 into its mature form and plays both beneficial and detrimental roles in the progression of cancer. In B16 melanoma models IL-18 acts as both an immunosuppressive and prometastatic factor. In an intravenous model of B16 melanoma IL-18 was shown to upregulate PD-1 expression on NK cells. Knock-down of IL-18 led to reduced pulmonary metastasis and increased NK cell function (94). Other studies showed that administration of IL-18 binding protein, which blocks IL-18, before injection of B16 melanoma into the spleen reduced metastasis to the liver. Mechanistically it was shown that treatment with IL-18 binding protein also reduced adhesion of B16 melanoma cells to hepatic sinusoidal endothelial cells leading to decreased metastasis (95). In concert with these data, administration of exogenous IL-18 led to an increase of adherent melanoma cells to sinusoidal endothelial cells (96). In humans an increase in IL-18 is correlated with various types of cancer including ovarian carcinoma, head and neck squamous carcinoma, breast cancer, and others (97–100). These data clearly demonstrate a pro-tumorigenic role of IL-18 both in humans and mice. Additionally, IL-18 may serve as a valuable biomarker for certain types of cancer.

In contrast, IL-18 plays a protective role during development of AOM-DSS CAC as mentioned above. *IL-18*^−/−^ and *IL-18r*^−/−^ mice were more susceptible to colon polyp formation and treatment of *caspase-1*^−/−^ mice with recombinant IL-18 leads to amelioration of disease (14, 70, 71). It was also shown that administration of IL-18 induced anti-tumor immunity in mice bearing B16 melanoma tumors expressing B7-1 (CD80). However administration of IL-18 to mice bearing B16 melanoma tumors alone had no effect on tumorigenesis (101). Therefore the role of IL-18 may vary depending on the types of tumor and the therapies it is combined with.

## Conclusion

It is clear that NLRs possess roles far from just pathogen recognition. NLRs play crucial roles in both promoting and dampening inflammation associated with tumors. Although the role of NLRs is best characterized in colorectal cancer, NLRs likely play a role in many other types of cancer. Further research needs to be performed to determine what specifically in the tumor environment leads to activation or down-regulation of NLRs. Danger signals released from dying cells have been shown to activate the NLRP3 inflammasome and it is possible that cancer cells release specific ligands capable of activating other NLRs. As some NLRs, like NLRP12, seem to be regulators of inflammation it could be hypothesized that mutations in these genes could correlate with tumor initiation and progression. A deeper understanding of the role of NLRs in the stages of cancer from initiation to metastasis will aid the development of new therapeutic strategies.

## Conflict of Interest Statement

The authors declare that the research was conducted in the absence of any commercial or financial relationships that could be construed as a potential conflict of interest.
